# Transcriptomic Analysis Reveals Core Genes and Mechanisms Linking Grilled Food Toxicants (4‐HNE, BaP, PhIP) to NASH‐HCC Progression

**DOI:** 10.1002/fsn3.72303

**Published:** 2026-08-31

**Authors:** Bingbing Qin, Ye Zhang, Na Li, Jing Li, Yuehua Luo, Xi Tang, Ruisheng Zhou, Tianqi Cui, Ying Tang

**Affiliations:** ^1^ Guangzhou University of Chinese Medicine Guangzhou Guangdong China; ^2^ Artemisinin Research Center Guangzhou University of Chinese Medicine Guangzhou Guangdong China; ^3^ School of Basic Medical Sciences Guangzhou University of Chinese Medicine Guangzhou Guangdong China; ^4^ Science and Technology Innovation Center Guangzhou University of Chinese Medicine Guangzhou Guangdong China; ^5^ The First Affiliated Hospital of Guangzhou University of Chinese Medicine Guangzhou Guangdong China; ^6^ Institute of Tumor Guangzhou University of Chinese Medicine Guangzhou Guangdong China

**Keywords:** computational toxicology, grilled food toxicants, machine learning, NASH‐HCC, transcriptomic analysis

## Abstract

Grilled food toxicants 4‐hydroxynonenal (4‐HNE), benzo[a]pyrene (BaP), and 2‐amino‐1‐methyl‐6‐phenylimidazo[4,5‐b]pyridine (PhIP) pose health risks, including non‐alcoholic steatohepatitis (NASH) and hepatocellular carcinoma (HCC). However, their synergistic molecular mechanisms in driving NASH to NASH‐HCC progression remain unclear. We employed network toxicology (CTD, GeneCards), bioinformatics analysis of the GSE164760 dataset, machine learning (LASSO, SVM‐RFE, Random Forest), SHAP interpretability analysis, immune infiltration profiling (CIBERSORT), miRNA‐TF‐mRNA network construction, and molecular docking to identify core genes and elucidate mechanisms. Toxicity prediction confirmed significant hepatotoxicity for all three compounds. Intersection analysis identified 8 key genes (InterGenes) enriched in oxidant detoxification, fatty acid metabolism, and chemical carcinogenesis pathways. Machine learning refined this to 6 CoreGenes (*GSTA1, EPHX1, CYP2E1, GPX3, ALB, TF*). SHAP analysis revealed that high expression of ALB, TF, GSTA1 and CYP2E1, together with low expression of GPX3 and EPHX1, correlated with increased progression risk. A CoreGenes‐based diagnostic nomogram achieved exceptional performance (AUC = 0.967). Immune infiltration analysis revealed significant shifts in regulatory T cells and M2 macrophages during progression. Molecular docking confirmed strong binding affinities between the toxicants and CoreGenes proteins (e.g., BaP‐GSTA1: −10.7 kcal/mol). Functional enrichment implicated dysregulated fatty acid metabolism, PPAR, and TGF‐beta signaling. This study identifies GSTA1, EPHX1, CYP2E1, GPX3, ALB, and TF as pivotal biomarkers and mediators through which 4‐HNE, BaP, and PhIP synergistically promote NASH‐HCC progression via oxidative stress, metabolic dysfunction and remodeling of the immune microenvironment. The CoreGenes signature and multi‐gene model provide a powerful tool for risk assessment and early intervention.

## Graphical abstract

This study identifies a six‐gene signature (GSTA1, EPHX1, CYP2E1, GPX3, ALB, TF) that mediates how grilled food toxicants (4‐HNE, BaP, PhIP) synergistically drive NASH‐to‐HCC progression via oxidative stress, metabolic dysfunction, and immune microenvironment remodeling, providing a robust diagnostic nomogram for early risk stratification.

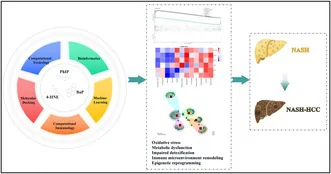

## 1 Introduction

The generation of toxic compounds during high‐temperature cooking (e.g., barbecuing) is a pivotal factor influencing food safety and environmental health. Among the identified byproducts, 4‐Hydroxynonenal (4‐HNE), benzo[a]pyrene (BaP), and 2‐Amino‐1‐methyl‐6‐phenylimidazo(4,5‐b) pyridine (PhIP) are prevalent in grilled foods, arising from lipid peroxidation and incomplete organic matter combustion (Sumer and Oz 2023). High concentrations of 4‐HNE, BaP, and PhIP have been associated with oxidative stress, DNA damage, and increased cancer risk (Liou et al. 2025; Wen et al. 2026). 4‐HNE induces cellular toxicity and disrupts homeostasis (Romani et al. 2023; Zhang et al. 2023). BaP is a potent mutagen and carcinogen, particularly impacting the liver (Liu et al. 2022; Wang et al. 2023). PhIP, one of the most abundant heterocyclic aromatic amines (HAAs) in cooked meat, is linked to colorectal and prostate cancer development (Nguyen et al. 2022; Wen et al. 2026; Zouiouich et al. 2025). Currently, the toxic side effects of by‐products from food processing and food additives have attracted societal attention. Despite the fact that food safety and environmental protection agencies have instituted pertinent regulatory standards, the persistent presence and bioaccumulation of 4‐HNE, BaP, and PhIP in grilled meat products and during the barbecuing process continue to raise concerns about their safety and potential health risks.

4‐HNE is an α,β‐unsaturated hydroxyalkenal produced by lipid peroxidation in cells, primarily derived from the oxidation of ω‐6 polyunsaturated fatty acids. In grilled foods, linoleic acid oxidation forms hydroperoxyoctadecadienoic acids (HPODE), which degrade to yield 4‐HNE (Błaszczyk and Karwowski 2025; Yang, Li, et al. 2023). 4‐HNE causes cytotoxic effects, particularly in liver cells (Riahi et al. 2010). At low concentrations, it acts as a pro‐fibrotic stimulus, promoting hepatic stellate cell (HSC) activation and extracellular matrix (ECM) deposition (Wang et al. 2026). Higher concentrations cause significant toxicity and hepatocyte death.

BaP is one of the polycyclic aromatic hydrocarbons and the primary carcinogenic substance produced in grilled foods. It is classified as a Group 1 carcinogen by the International Agency for Research on Cancer (IARC). Research suggests BaP exposure links non‐alcoholic steatohepatitis (NASH) and hepatocellular carcinoma (HCC) through cellular metabolic dysfunction and disrupted DNA damage repair (Yang, Jin, et al. 2023; Yang, Li, et al. 2023). BaP exacerbates DNA damage by inhibiting DNA repair enzyme expression (Caiment et al. 2015), leading to chronic liver inflammation and fibrosis.

PhIP, an HAA formed during high‐temperature cooking, can induce breast, colon, and prostate cancers in rodents. Its hepatic metabolism and toxicity are confirmed. PhIP forms DNA adducts in the liver, causing DNA damage and elevated cancer risk. Its liver toxicity is also associated with oxidative stress and cellular damage. PhIP induces hepatic enzymes like CYP2E1 and CYP1A2, leading to lipid peroxidation, mitochondrial dysfunction, and hepatotoxicity (Cao et al. 2023).

NASH represents a progressive liver disease with a significantly elevated risk of HCC, driven by complex multi‐level pathophysiological interactions. Progressive metabolic dysfunction fosters a procarcinogenic microenvironment, predominantly mediated through chronic inflammation, ultimately culminating in end‐stage liver diseases, including HCC and liver failure (Xi et al. 2025). Prolonged chronic inflammation ultimately leads to HCC. Evidence indicates 4‐HNE, BaP, and PhIP exacerbate liver damage and fibrosis by activating inflammatory pathways (e.g., NF‐κB, AP‐1) and promoting apoptosis escape (Li et al. 2024; Souza et al. 2016). Although these compounds are established hepatotoxins and carcinogens, their precise synergistic mechanisms in orchestrating the transition from NASH to HCC warrant further mechanistic investigation.

This study combines network toxicology, bioinformatics, and machine learning to systematically investigate the role of 4‐HNE, BaP, and PhIP in promoting the progression and carcinogenesis of NASH. Molecular docking was employed to assess the binding patterns of these compounds with core target proteins, shedding light on the potential molecular mechanisms driving disease progression. Our findings establish a theoretical framework for preventing grilled food‐associated NASH and NASH‐HCC. It also offers an innovative method for assessing carcinogenic risks of food processing byproducts, with significant implications for public health protection and environmental policy.

## 2 Methods

### 2.1 Toxicity Analysis and Target Gene Collection

The chemical name and structural characteristics of 4‐HNE, BaP, and PhIP were extracted from the PubChem database (https://pubchem.ncbi.nlm.nih.gov/). Subsequently, the toxicity verification was conducted by using the CompTox (https://comptox.epa.gov/dashboard/), ADMETlab 2.0 (https://admetmesh.scbdd.com/), and ProTox‐3.0 (https://tox.charite.de/). Potential human target genes were identified from the CTD database (http://ctdbase.org/) and GeneCards (https://www.genecards.org/).

### 2.2 Acquisition of GEO Data and Identification of Differentially Expressed Genes

GSE164760 dataset (platform: GPL13667) from the GEO database (https://www.ncbi.nlm.nih.gov/geo/) was used for analysis, which includes 53 NASH‐HCC and 74 NASH samples. The “limma” R package was applied with the parameter setting of *p* < 0.05 and |Log_2_FC| > 1 to obtain differentially expressed genes (DEGs). Then, we employed the “ggpubr” and “pheatmap” packages to visualize the results and draw volcano plots and heat maps.

### 2.3 Identification of InterGenes and Functional Enrichment Analysis

The target genes of the three compounds, 4‐HNE, BaP, and PhIP, were identified based on the “VennDiagram” R package, and were then intersected with DEGs to obtain the intersection genes (InterGenes) for downstream analysis. We employed R packages of “clusterProfiler,” “enrichplot,” and “ggplot2” to perform Gene Ontology (GO) functional annotation analysis on InterGenes, as well as Kyoto Encyclopedia of Genes and Genomes (KEGG) pathway enrichment analysis and visualization. The selection of results with statistical significance was executed using a threshold of *p*. adjust < 0.05.

### 2.4 Machine Learning—Validation of InterGenes

To identify key genes associated with toxicity, three machine learning approaches—Random Forest (RF), Support Vector Machine (SVM), and Least Absolute Shrinkage and Selection Operator (LASSO) were employed to screen the Intergenes. By taking the intersection of the genes selected from the three aforementioned methods, the final set of core genes was established. We utilized the Receiver operating characteristic (ROC) curve analysis to assess the independent diagnostic capability of each core gene. Moreover, a multi‐gene logistic regression model was constructed based on the core genes, and then we evaluated the model.

### 2.5 SHAP Analysis

To quantify the feature contribution of machine learning models and enhance the interpretability of prediction outcomes, we adopted the SHapley Additive exPlanations (SHAP) analysis method, which is based on the Shapley value in cooperative game theory. The subsequent analysis of the output results of machine learning models based on SHAP analysis facilitates the identification of key toxicity targets and the exploration of their mechanism.

### 2.6 Construction of Protein–Protein Interaction Network and Enrichment Analysis

We then proceeded to analyze protein–protein interaction (PPI) between core genes (CoreGenes) using the STRING database (https://string‐db.org/), setting the confidence score threshold to ≥ 0.4. Concurrently, to elucidate the biological functions of CoreGenes, we performed Gene Set Enrichment Analysis (GSEA).

### 2.7 Construct the CoreGenes' Risk Prediction Model and Conduct Diagnostic Validation

The GSE164760 dataset was utilized to analyze the expression trends of the CoreGenes in NASH and NASH‐HCC samples. To further validate the clinical applicability of the CoreGenes, we used the “rms” R package to construct the predictive nomogram. The accuracy and reliability of the model were validated by using calibration curves, area under the curve (AUC) value, and decision curve analysis (DCA). Furthermore, the Bootstrap method was employed for the internal validation of the dataset to ensure the model's stability and generalizability.

### 2.8 External Validation of CoreGenes Expression and Risk Model

To validate the association of the six CoreGenes with disease progression and assess the generalizability of our diagnostic model, we performed external validation using the independent GSE162694 cohort, which includes NASH patients with fibrosis stage (F0–F4) and NAFLD Activity Score (NAS) annotations. The risk score for each sample was calculated using the logistic regression coefficients derived from the training set (GSE164760). Model performance was evaluated by comparing risk scores between mild (F0–F1) versus advanced (F3–F4) fibrosis and low (NAS 0–1) versus high (NAS 6–7) disease activity, followed by ROC analysis.

### 2.9 Immune Infiltration Analysis

The CIBERSORT (Cell‐type Identification by Estimating Relative Subsets of RNA Transcripts) deconvolution algorithm analysis yielded the identification of 22 distinct types of human immune cells, with the quantification of their relative proportions in each sample performed using a threshold of *p* < 0.05. Subsequently, an analysis was conducted to examine the correlation between the CoreGenes' expression and immune cell infiltration abundance.

### 2.10 Association of CoreGenes With Immune Checkpoint Expression and Immunotherapy Response

To explore the link between CoreGenes and immunotherapy response in NASH‐HCC, we performed Spearman correlation analysis between the six CoreGenes and key immune checkpoints using NASH‐HCC samples from the GSE164760 dataset. The Immunophenotype Score (IPS), reflecting overall immune activity, was calculated using the IOBR package. Samples were stratified into high and low expression groups based on the median expression of each CoreGenes, and IPS differences were assessed using the Wilcoxon test.

### 2.11 Construction of miRNA‐TF‐mRNA Interaction Network of CoreGenes

By integrating the miRBD (http://www.mirdb.org/) and TargetScan databases (https://www.targetscan.org/) to identify miRNAs of CoreGenes. Additionally, transcription factors corresponding to CoreGenes were obtained from the hTFtarget (https://guolab.wchscu.cn/hTFtarget) and the KnockTF database (http://www.licpathway.net/KnockTFv2/index.php). Using R language software, we visualized the miRNA‐TF‐mRNA regulatory network based on miRNA, TF, and mRNA data, which revealed the potential molecular mechanisms by which 4‐HNE, BaP, and PhIP promote NASH progression and carcinogenesis.

### 2.12 Molecular Docking

The 3D structures of the genes were downloaded from the Protein Data Bank (PDB) website (https://www.rcsb.org/) and the Uniprot website (https://www.uniprot.org/). The SDF structures of the molecules were downloaded from the PubChem database (https://pubchem.ncbi.nlm.nih.gov/). Finally, the molecular docking was completed using the CB‐Dock2 website (https://cadd.labshare.cn/cb‐dock2/php/index.php).

## 3 Results

### 3.1 Toxicity Assessment and Target Gene Acquisition

As shown in Table 1, the foodborne contaminants 4‐HNE, BaP, and PhIP exhibit distinct physicochemical and toxicological properties, highlighting their potential risks to food safety and human health. BaP, a polycyclic aromatic hydrocarbon commonly found in grilled and smoked foods, demonstrates high persistence due to its extreme hydrophobicity and strong soil adsorption, suggesting a tendency to accumulate in fatty food matrices. In contrast, 4‐HNE and PhIP show greater mobility in food systems due to their moderate solubility and shorter half‐lives. All three compounds are classified as toxicity category 4, with BaP posing the highest dietary exposure risk and PhIP exhibiting the strongest hepatotoxic potential. Although 4‐HNE's balanced properties may enhance its bioavailability in both food and biological systems. Therefore, further investigation into these compounds' toxicological effects in humans is warranted.

**TABLE 1:** Environmental and toxicological profiles of 4‐HNE, BaP, and PhIP.

Parameter	Unit	4‐HNE	BaP	PhIP
**Physicochemical properties**				
Molecular weight	g/mol	156.23	252.32	224.27
Water solubility	mol/L	4.43 × 10^−2^	8.40 × 10^−9^	1.95 × 10^−3^
LogKow	—	1.79	6.13	2.23
**Environmental fate**				
Soil adsorption (Koc)	L/kg	123	8.91 × 10^5^	129
Biodegradation half‐life	days	4.47	224	3.98
Vapor pressure	mmHg	1.46 × 10^−2^	3.49 × 10^−9^	4.65 × 10^−8^
Atmospheric hydroxylation rate	cm^3^ molecule^−1^ s^−1^	4.27 × 10^−11^	7.76 × 10^−11^	5.37 × 10^−11^
**Exposure and toxicity**				
Human exposure (95th %ile)	mg/kg/day	5.17 × 10^−4^	1.32 × 10^−2^	6.94 × 10^−5^
LD50 (oral)	mg/kg	1925	316	500
Human hepatotoxicity	Probability	0.56	0.739	0.871
Ecotoxicity	Activity (Probability)	Active (0.57)	Active (0.91)	Active (0.56)
Predicted toxicity class	—	4	4	4

*Note:* Koc: Soil organic carbon‐water partition coefficient (measure of soil adsorption potential). LogKow: Octanol–water partition coefficient (indicator of hydrophobicity). 95th %ile: 95th percentile of estimated daily human exposure. LD50: Median lethal dose (oral administration in animal models).

Systematic searches of the CTD and Genecards databases identified 1002 human target genes for 4‐HNE, 19,915 for BaP, and 4977 for PhIP (Table S1).

### 3.2 Identification of InterGenes and Functional Enrichment Analysis

**FIGURE 1:**
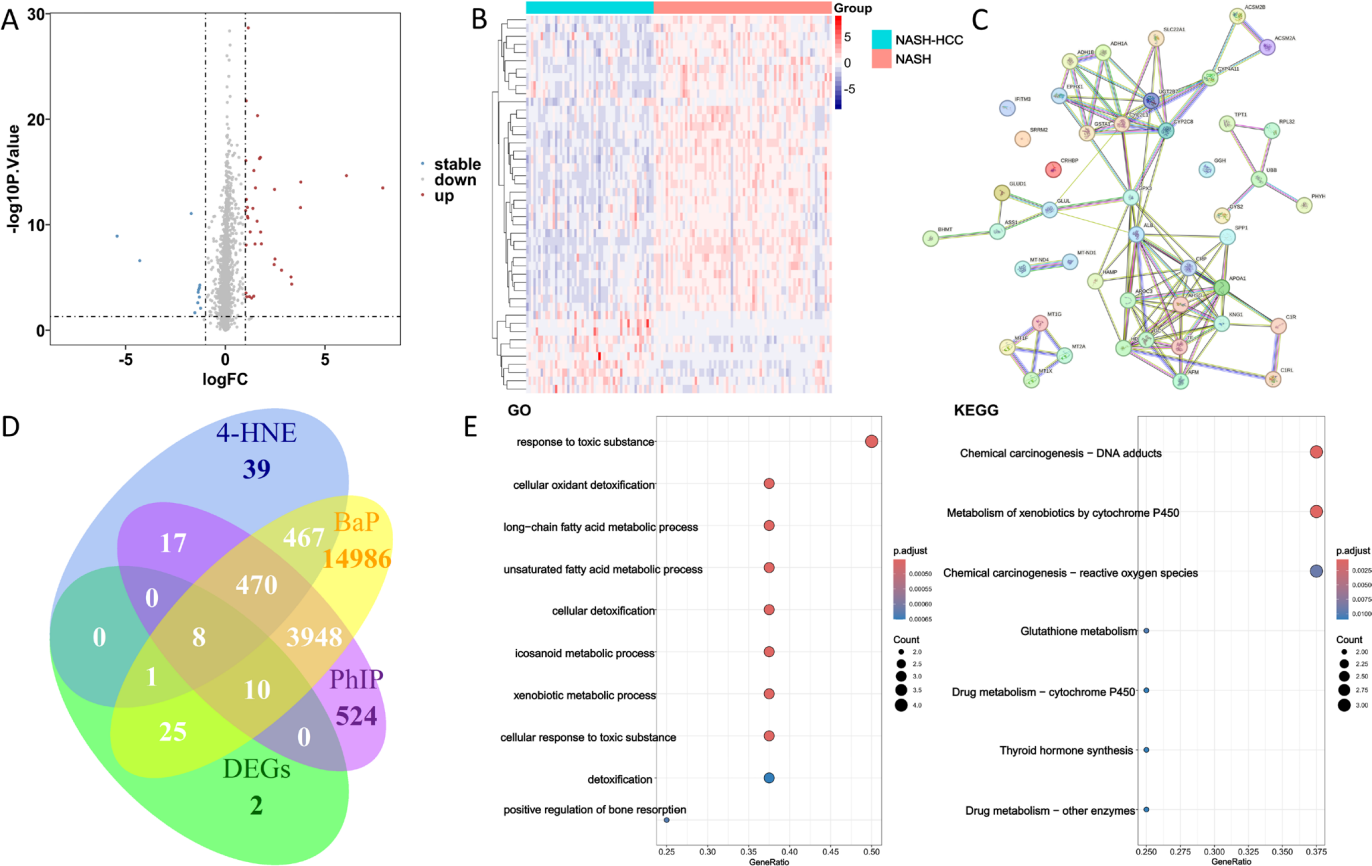
Hub gene identification and functional enrichment analysis. (A) Volcano plot of DEGs in the GSE164760 dataset. (B) Heatmap of DEGs in the GSE164760 dataset. (C) Protein interaction network of DEGs. (D) Venn diagram of the targets of network toxicity and DEGs. (E) GO and KEGG enrichment analysis of InterGenes.

Differential gene expression analysis of the GSE164760 dataset identified 46 DEGs, comprising 36 upregulated and 10 downregulated (Table S2). These DEGs were visualized using volcano plots (Figure 1A) and heatmaps (Figure 1B). A PPI network was constructed for the DEGs (Figure 1C; Table S3). Intersection analysis between the DEGs and the compounds' target genes identified eight overlapping genes, termed InterGenes (Figure 1D). GO and KEGG enrichment analysis of the InterGenes revealed significant associations with cellular oxidant detoxification, response to toxic substances, fatty acid metabolic process, chemical carcinogenesis (DNA adducts and reactive oxygen species), and glutathione metabolism (Figure 1E).

### 3.3 Identification of CoreGenes Through Machine Learning

**FIGURE 2:**
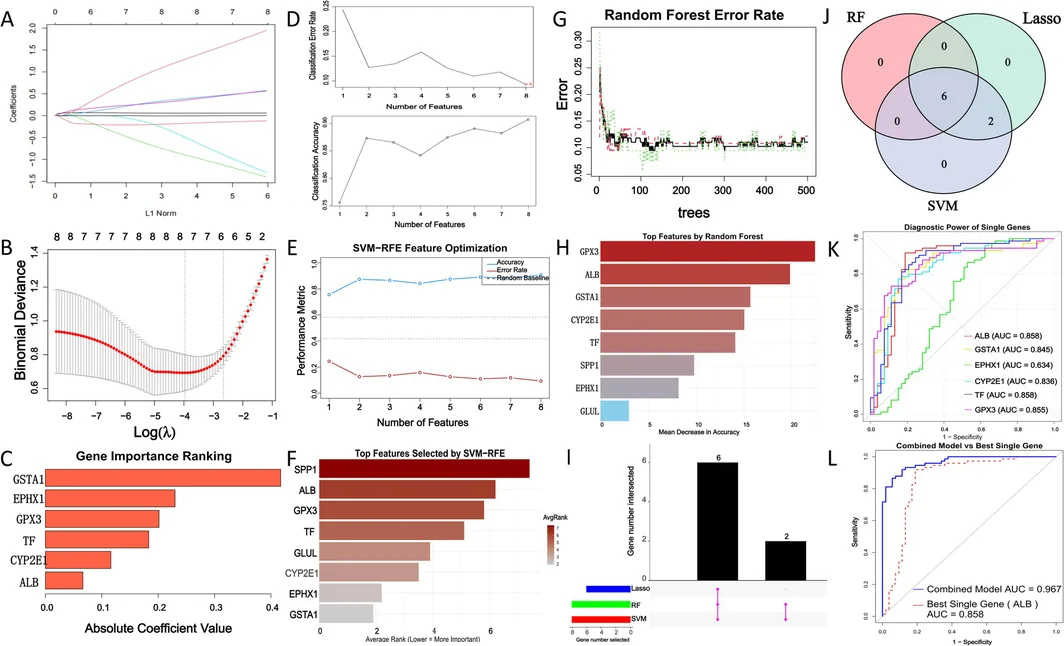
Identification of CoreGenes through machine learning. (A) LASSO coefficient path plot. (B) LASSO binomial deviance curve. (C) LASSO feature importance plot. (D) SVM‐RFE cross‐validation error curve and accuracy curve. (E) SVM‐RFE plot depicting the relationship between feature number and performance. (F) SVM‐RFE feature importance plot. (G) Random forest error rate plot. (H) Random forest feature importance plot. (I) Upset plot of machine learning intersection. (J) Venn diagram of CoreGenes acquisition. (K) Single‐gene diagnostic model evaluation. (L) CoreGenes combined diagnostic model evaluation.

To further refine the candidate gene list and identify the most pivotal biomarkers, we employed LASSO (Figure 2A–C), SVM‐RFE (Figure 2D–F), and RF (Figure 2G,H). Integrating these results identified six consensus genes (GSTA1, EPHX1, CYP2E1, GPX3, ALB, and TF), designated as CoreGenes (Figure 2I,J) (Table S4). ROC curve analysis showed diagnostic value for each gene, with ALB and TF having the highest AUC (AUC = 0.858) (Figure 2K). A multi‐gene logistic regression model based on the CoreGenes achieved an AUC of 0.967, significantly outperforming the best single genes ALB and TF, validating the multi‐gene combination strategy (Figure 2L). Together, these results demonstrate that the integration of multiple machine learning approaches successfully identified a robust and highly diagnostic core genes.

### 3.4 SHAP Explanatory Analysis

**FIGURE 3:**
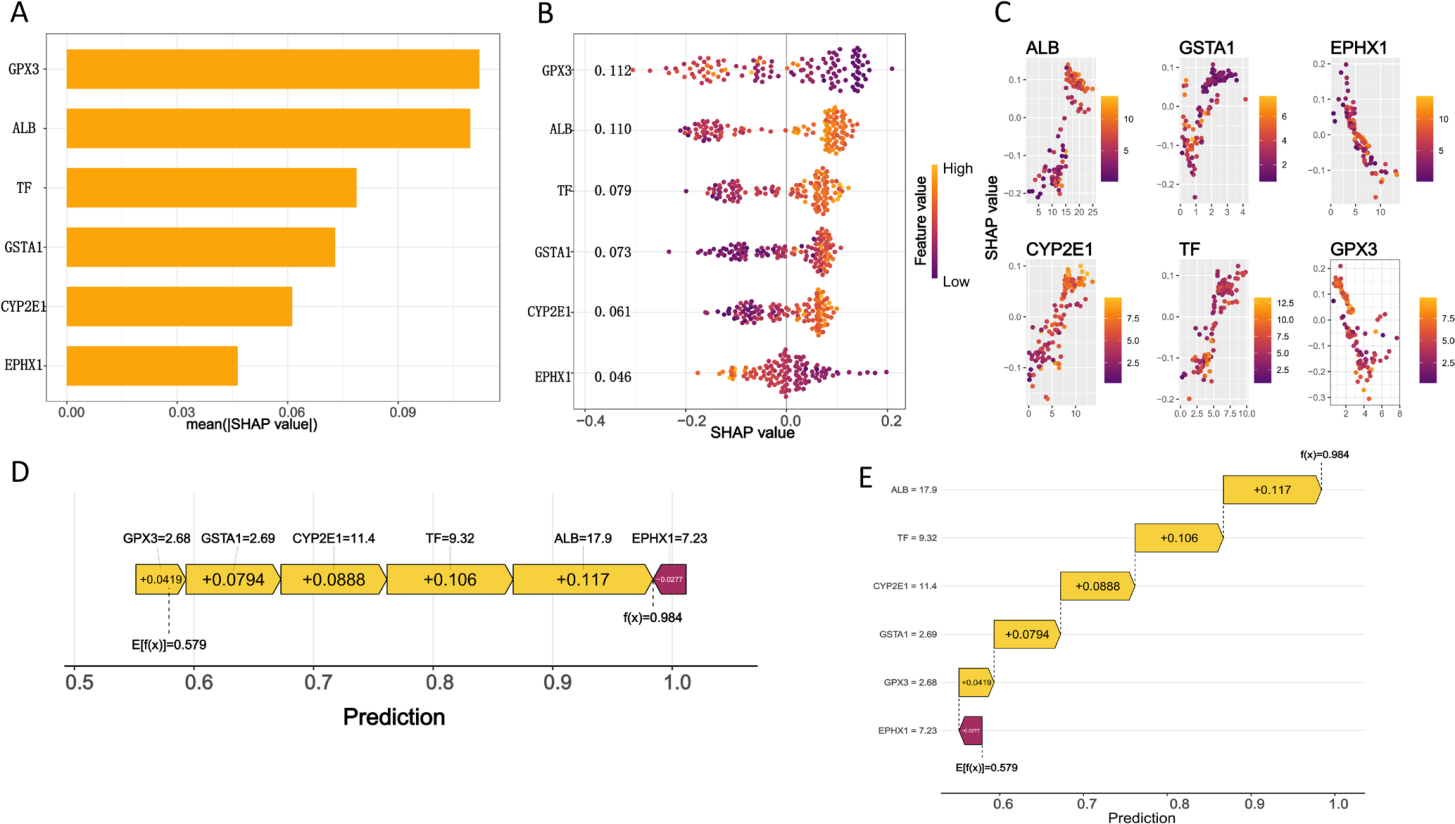
SHAP explanatory analysis. (A) Bar plot showed the contribution magnitudes of six genes in the random forest model. (B) Beeswarm plot highlighted the influence of genes at the sample level. (C) Scatter plot illustrated the correlation between CoreGenes and SHAP values. (D) Force plot presented individual sample classification based on integrated prediction. (E) Waterfall plot presenting cumulative contribution of CoreGenes to individual sample prediction scores.

To elucidate the model's mechanism, SHAP analysis was applied using the “shapviz” R package, identifying and ranking the core genes based on their contribution magnitude. The ensuing analysis yielded results that were presented through multidimensional visualization, incorporating bar charts (feature importance), swarm plots (SHAP value distribution), scatter plots (feature interactions), force plots (individual prediction explanations), and waterfall plots (cumulative contributions) (Figure 3A–E).

The bar chart indicates GPX3 and ALB contribute most significantly to the model, while EPHX1 has weaker influence (Figure 3A). Furthermore, high expression of ALB, TF, GSTA1, and CYP2E1, along with low expression of GPX3 and EPHX1, has been associated with a higher disease progression probability (Figure 3B). The scatter plot indicates positive correlations between SHAP values and expression levels of ALB, TF, GSTA1, and CYP2E1, suggesting higher expression increases predictive value. Conversely, SHAP values negatively correlate with GPX3 and EPHX1 expression, indicating increased expression reduces predictive value (Figure 3C). These findings suggest core gene expression changes may link to NASH‐to‐NASH‐HCC progression risk. Force plots display each gene's independent contribution direction and magnitude (Figure 3D), whereas waterfall plots quantify core genes' cumulative contribution to individual sample prediction scores (Figure 3E). Analysis of a representative NASH sample shows ALB, TF, GSTA1, GPX3, and CYP2E1 act as primary positive regulators, increasing the predictive score, while EPHX1 serves as the primary negative regulator, decreasing it.

Collectively, the SHAP analysis not only validated the importance of the CoreGenes identified by the feature selection algorithms but also provided mechanistic insights into their roles in disease progression, with specific genes exhibiting promotive or protective effects.

### 3.5 Construction of the PPI Network and Gene Set Enrichment Analysis of CoreGenes

**FIGURE 4:**
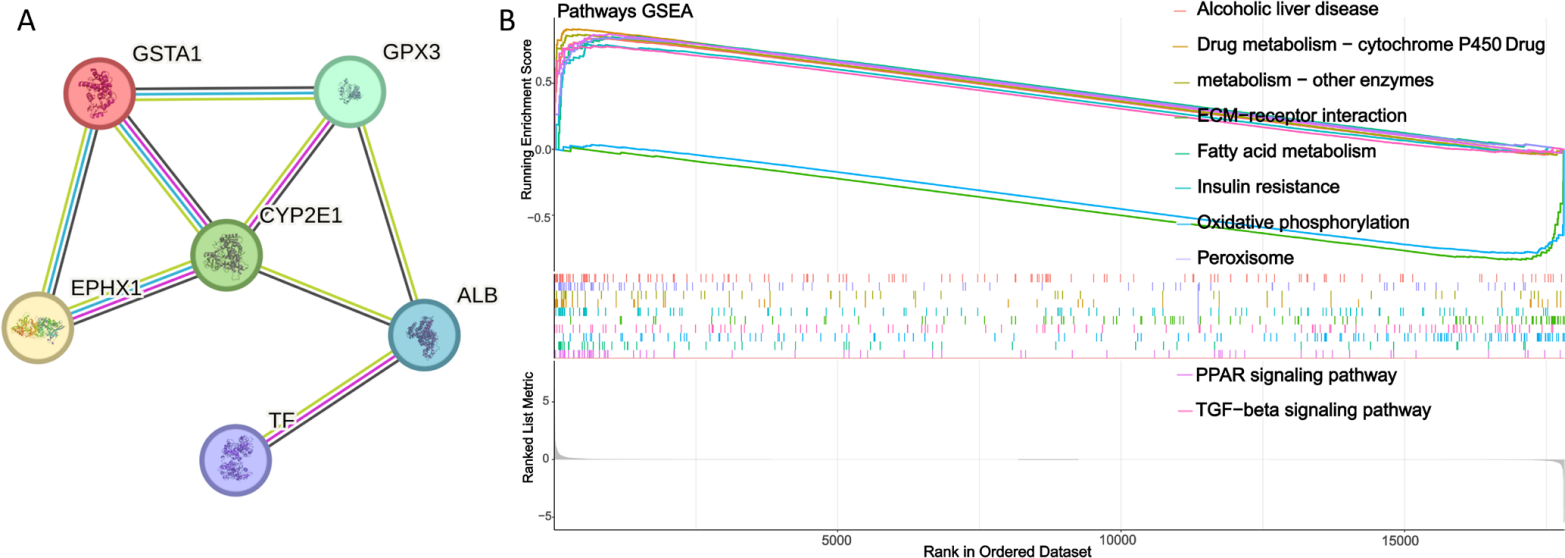
PPI network and GSEA of coregenes. (A) PPI network of coregenes. (B) GSEA of coregenes.

To investigate the functional associations and underlying biological pathways of the six CoreGenes, we performed a PPI network analysis and GSEA. PPI analysis of the core genes using the STRING database showed that the six genes form functional modules through interactions, potentially regulating shared pathways and supporting their synergistic role in NASH to NASH‐HCC progression (Figure 4A). GSEA functional enrichment analysis revealed significant associations with alcoholic liver disease, drug metabolism, ECM‐receptor interaction, fatty acid metabolism, insulin resistance, oxidative phosphorylation, PPAR signaling, and TGF‐beta signaling pathways (Figure 4B). These analyses collectively suggest that the CoreGenes do not function in isolation but rather as a coordinated network, crucially implicated in the metabolic and pro‐fibrotic inflammatory processes that drive NASH and its progression to HCC.

### 3.6 Construction of Risk Prediction Model and Diagnostic Validation of the CoreGenes

**FIGURE 5:**
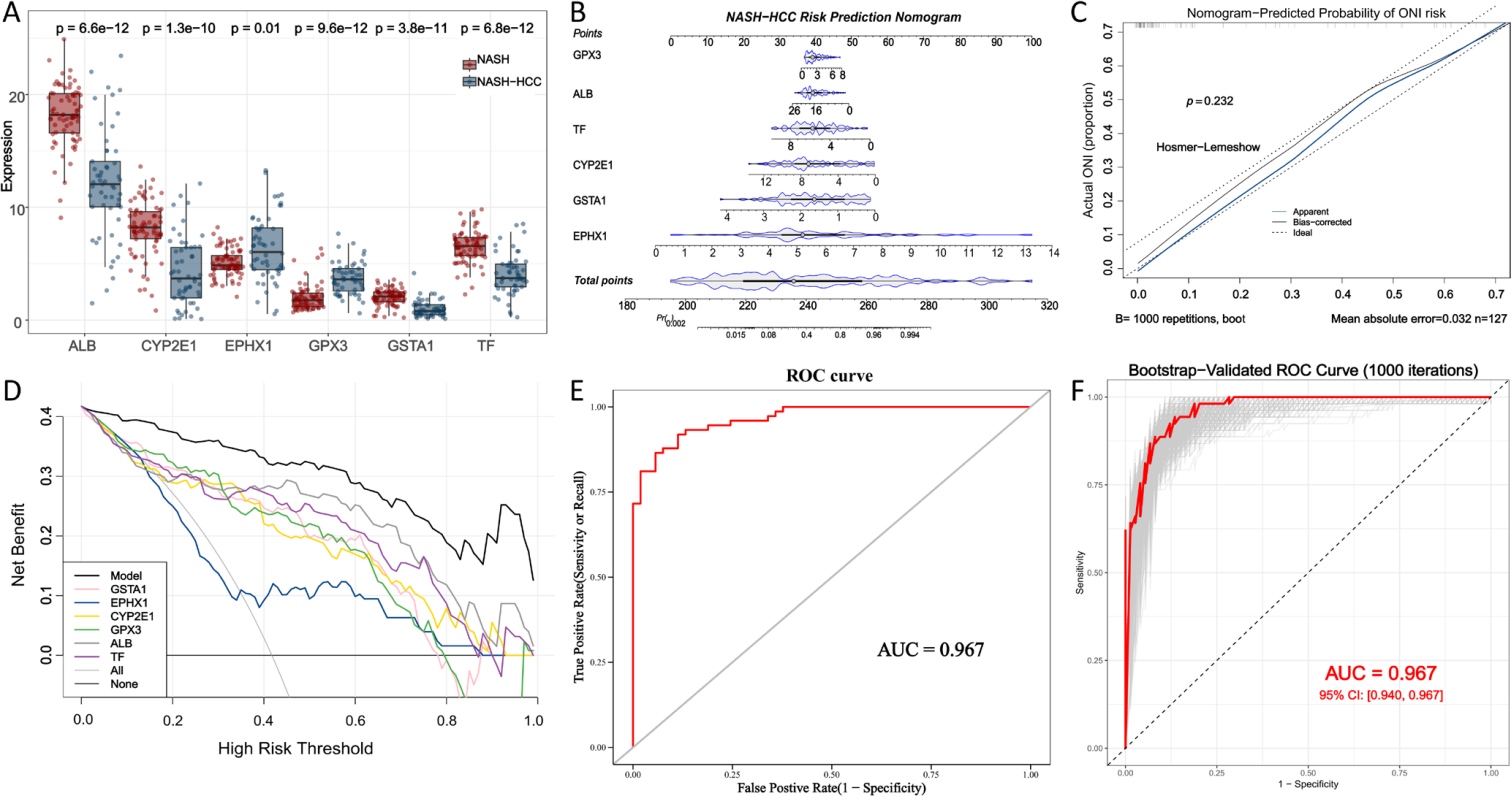
Construction of risk prediction model and diagnostic validation of the CoreGenes. (A) Expression of CoreGenes of GSE164760. (B) Nomogram predicting the risk in the training set based on CoreGenes. (C) Calibration curve for the accuracy of the line chart prediction: The higher the convergence of the three curves, the better alignment between the predicted and the actual probabilities. (D) DCA of the column chart's benefits: When the model curve is above the ALL and None curves, it indicates superior decision‐making performance. (E) ROC curve assessment of the diagnostic performance of feature genes in the dataset. (F) Internal validation of the dataset using Bootstrap resampling.

The expression levels of CoreGenes in NASH and NASH‐HCC samples from the GSE164760 dataset were analyzed. Among these, ALB, CYP2E1, GSTA1, and TF were underexpressed in NASH‐HCC samples, whereas EPHX1 and GPX3 were overexpressed in NASH‐HCC samples (Figure 5A). To enhance clinical utility and assess NASH‐to‐NASH‐HCC progression risk, a diagnostic nomogram was developed using the six CoreGenes, quantifying progression risk through expression‐based scoring (Figure 5B). The calibration curve demonstrated strong agreement with the ideal line (Hosmer‐Lemeshow test *p* = 0.232; average absolute error 0.032), indicating robust clinical calibration (Figure 5C). Furthermore, the decision curve demonstrated that the joint model‐guided decision yielded clinical advantages for patients in predicting the risk of progression from NASH to NASH‐HCC (Figure 5D). The black curve of the model always lies above the gray curve of ALL and the single gene curve, and the net benefit of the combined model is significantly higher than that of a single marker. ROC analysis reveals that the AUC value of the model for distinguishing NASH from NASH‐HCC is 0.967, indicating that the model possesses remarkably strong discriminatory capability and high accuracy (Figure 5E). Bootstrap resampling validation, with 1000 iterations, yielded an area AUC of 0.967, with a 95% confidence interval (CI) ranging from 0.940 to 0.967. This finding suggests that the model developed in this study exhibits both exceptional and stable predictive capabilities (Figure 5F). In summary, we successfully developed a CoreGenes‐based nomogram that demonstrates robust discriminatory power, excellent calibration, and substantial clinical net benefit, representing a reliable and translatable tool for risk stratification in the NASH‐HCC continuum.

### 3.7 External Validation of CoreGenes Expression and Risk Model

We performed independent validation using the GSE162694 cohort. As shown in Figure S1A–C, all six CoreGenes showed significant expression differences between mild (F0–1) and advanced (F3–4) fibrosis, with progressive changes across F0–F4 stages (all *p* < 0.05). Notably, EPHX1 showed decreased expression in advanced fibrosis compared to mild fibrosis in the GSE162694 cohort, which appears opposite to its upregulation in NASH‐HCC versus NASH in the GSE164760 dataset.

Validation of the risk model across fibrosis stages revealed significantly higher risk scores in advanced compared to mild fibrosis (*p* = 5 × 10^−5^), demonstrating good discriminatory ability with an AUC of 0.802 (Figure S1D). Risk model validation using the NAS further demonstrated that risk scores were significantly higher in the high NAS group (6–7) compared to the low NAS group (0–1) (*p* = 0.0012), with an AUC of 0.751 (Figure S1E).

Collectively, these results demonstrate that the six CoreGenes are significantly associated with fibrosis progression, and our risk model exhibits good generalizability for stratifying NASH patients by disease severity.

### 3.8 Immune Infiltration Analysis and Immune Cell Correlation Analysis

**FIGURE 6:**
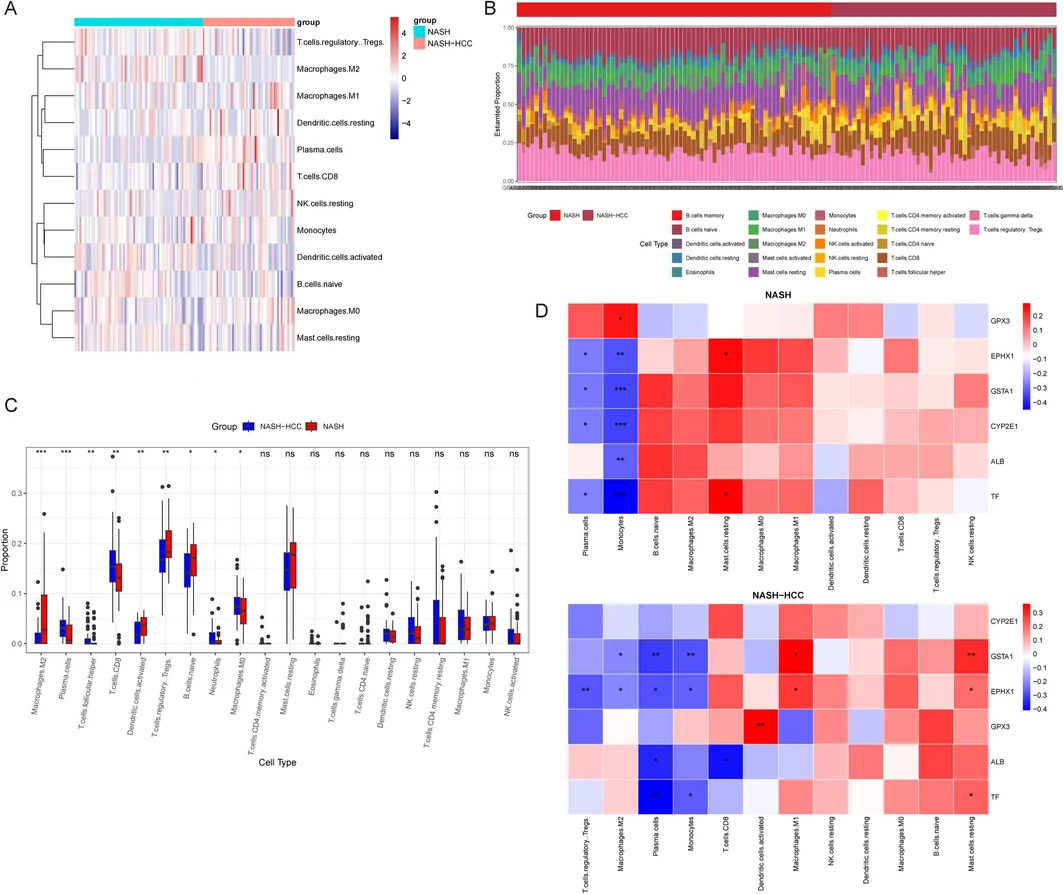
Immune infiltration analysis and immune cell correlation analysis. (A) Differences in the distribution of immune cells between disease groups. (B) Relative proportions of various types of immune cells between disease groups. (C) Proportional distribution of 22 immune cell subsets between disease groups. (D) Heatmap presented the correlations between CoreGenes and immune cells *p < 0.05, **p < 0.01, ***p < 0.001.

Given the critical role of the tumor immune microenvironment (TIME) in cancer progression, we characterized the immune cell infiltration landscape and its association with the CoreGenes in NASH and NASH‐HCC samples. We assessed immune cell distribution across sample groups using the CIBERSORT algorithm and GSE164760 dataset (Figure 6A,B). Compared to NASH samples, NASH‐HCC showed significantly altered infiltration levels in nine immune cell types (Figure 6C). The core gene‐immune cell association analysis revealed associations (Figure 6D), such as in NASH samples, where core genes were found to be associated exclusively with Plasma cells, Monocytes, and Mast cells resting. In contrast, in NASH‐HCC, these same core genes were found to be significantly associated with T cell regulatory, Macrophages M2, Plasma cells, Monocytes, T cells CD8, Dendritic cells activated, Macrophages M1, and Mast cells resting. These findings suggest that the CoreGenes are closely intertwined with the dynamic immune landscape, and their altered expression during the NASH‐to‐HCC transition may contribute to shaping an immunosuppressive microenvironment conducive to tumor progression.

### 3.9 Association of CoreGenes With Immune Checkpoint Expression and Immunotherapy Response in NASH‐HCC

To explore the potential link between CoreGenes and immunotherapy response in NASH‐HCC, we analyzed their correlation with key immune checkpoints in NASH‐HCC samples. Correlation analysis revealed that CoreGenes expression levels were significantly negatively correlated with PD‐L1 (CD274), PD‐1 (PDCD1), and CTLA‐4, with GSTA1 showing the strongest correlations (Figure S2A).

We further calculated the IPS, a transcriptome‐based metric reflecting overall immune activity. No significant association was observed between CoreGenes expression and IPS scores in NASH‐HCC samples (all *p* > 0.05) (Figure S2B).

### 3.10 miRNA‐TF‐mRNA Interaction Network of CoreGenes

**FIGURE 7:**
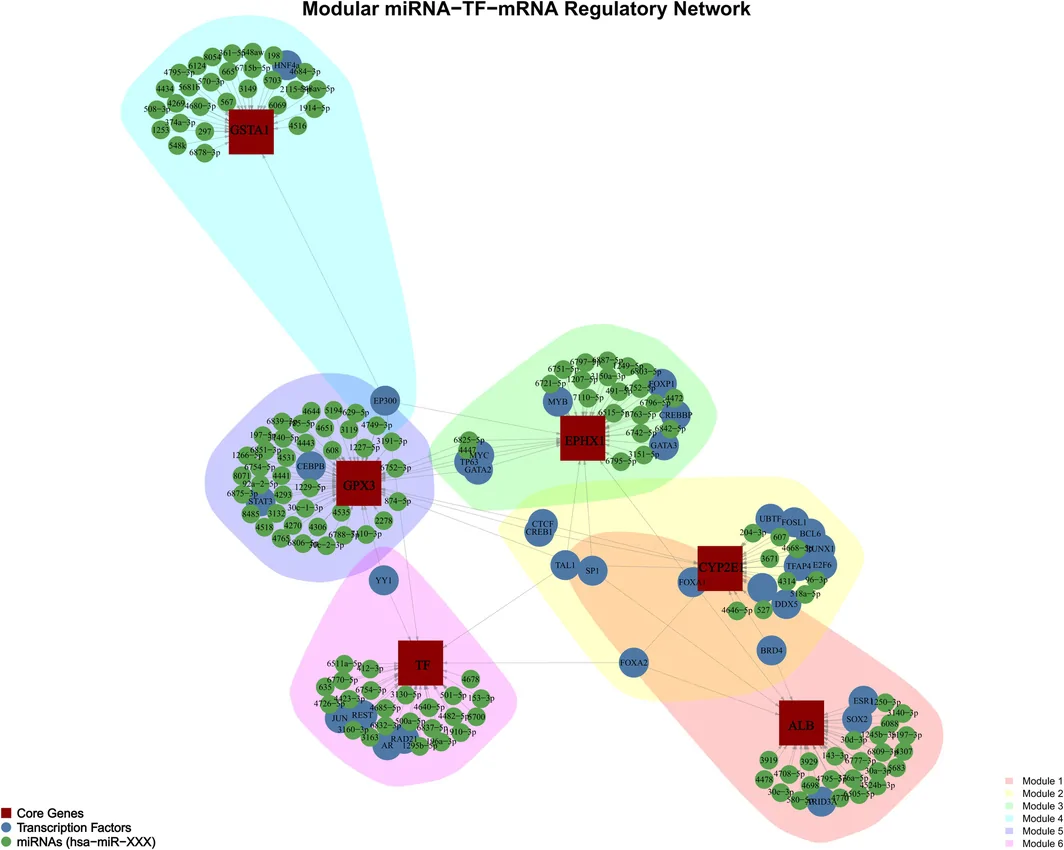
miRNA‐TF‐mRNA interaction network of CoreGenes.

Delving deeper into the regulatory hierarchy, we sought to identify the key transcription factors and microRNAs (miRNAs) that potentially orchestrate the expression of the six CoreGenes. To elucidate regulatory mechanisms, this study predicted microRNAs and transcription factors targeting the core genes. Based on R language visualization and integrated the regulatory network, the interactions between 146 miRNAs, 51 transcription factors, and six core genes were demonstrated (Figure 7) (Table S5). This preliminary regulatory map highlights potential upstream regulators for future functional validation and identifies novel candidate therapeutic targets for intervening in the disease process.

### 3.11 Molecular Docking

**FIGURE 8:**
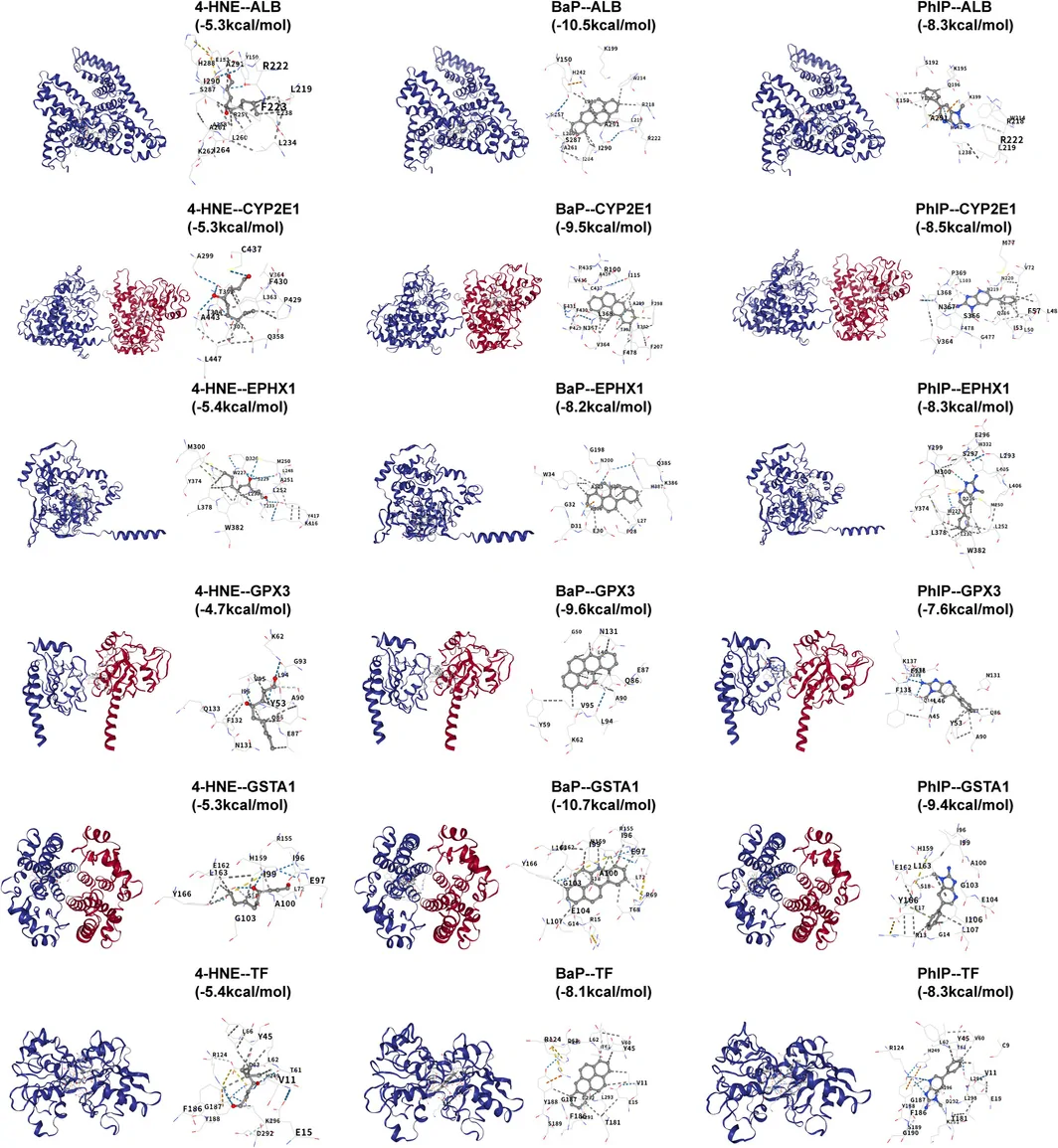
Molecular docking of 4‐HNE, BaP and PhIP to CoreGenes.

To further investigate the potential interactions among 4‐HNE, BaP, PhIP and CoreGenes, molecular docking simulations were conducted. Molecular docking demonstrated strong binding affinity between all three compounds and the six CoreGenes (Figure 8). BaP exhibited the highest binding energy with GSTA1 (−10.7 kcal/mol), whereas 4‐HNE showed the lowest binding energy with GPX3 (−4.7 kcal/mol). The highly significant binding results, particularly the strong interaction between BaP and GSTA1, suggest that these toxins may directly target CoreGenes, potentially contributing to liver damage and carcinogenesis.

## 4 Discussion

4‐HNE, BaP, and PhIP are compounds abundantly formed in grilled foods through lipid oxidation, protein thermal decomposition, and incomplete high‐temperature combustion, particularly in charcoal‐grilled meats, smoked products, and fried foods. Despite their popularity, these foods are a significant dietary source of these toxins, which have raised increasing health concerns. Studies have shown that 4‐HNE can activate the NF‐κB pathway, elevating pro‐inflammatory factors such as TNF‐α and IL‐1β, thereby exacerbating liver inflammation and fibrosis. As demonstrated in other studies, the accumulation of 4‐HNE is associated with poor prognosis in HCC, with low 4‐HNE levels linked to vascular invasion and poor tumor differentiation (Gerber et al. 2023). Meanwhile, BaP has been reported to promote liver cancer cells to secrete exosomes containing specific circular RNAs, which can convert normal lung fibroblasts into cancer‐associated fibroblasts, thereby facilitating metastatic spread (Mu et al. 2024). These findings reveal the hepatotoxicity of grilled food compounds, though long‐term low‐dose cumulative effects require further systematic evaluation. The precise mechanisms by which 4‐HNE, BaP, and PhIP contribute to the progression from NASH to NASH‐HCC also warrant deeper investigation.

This study assessed the hepatotoxicity of 4‐HNE, BaP, and PhIP using ProTox‐3.0 and ADMETlab 2.0. CTD and Genecards databases were used to identify human target genes for each compound. Analysis of the GSE164760 dataset revealed DEGs in NASH and NASH‐HCC. Integration of the compound target genes with the DEGs yielded a set of intersection genes. GO and KEGG enrichment implicated these compounds in NASH‐to‐NASH‐HCC progression via cellular oxidant detoxification, response to toxic substances, fatty acid metabolism, chemical carcinogenesis (DNA adducts and reactive oxygen species), and glutathione metabolism. NASH progression to HCC involves fatty acid metabolism, accelerated lipid synthesis, immunity, and microenvironment alterations. 4‐HNE, a major product of lipid peroxidation, contributes to hepatocarcinogenesis by inducing DNA oxidative damage and genomic instability (Yuan et al. 2025). It also promotes DNA damage and mutations by inhibiting antioxidant enzymes (e.g., ALDH2), increasing oxidative stress sensitivity and inducing hepatocellular carcinoma (Csala et al. 2015; Zhong and Yin 2015). BaP metabolites, which are its ultimate carcinogenic forms, cause DNA oxidative damage via the generation of reactive oxygen species (ROS). BaP modulates oxidative stress by influencing heat shock protein (HSP) and antioxidant enzyme expression, including SOD (Zheng et al. 2011), aligning with our findings.

Six CoreGenes (GSTA1, EPHX1, CYP2E1, GPX3, ALB, and TF) were identified through machine learning approaches. SHAP analysis revealed ALB and GPX3 contributed most significantly to the model. Elevated expression of ALB, TF, GSTA1, and CYP2E1, coupled with reduced expression of GPX3 and EPHX1, was correlated with a higher probability of disease progression. PPI network analysis demonstrated robust interactions among the CoreGenes. GSEA implicated 4‐HNE, BaP, and PhIP in the progression from NASH to HCC via pathways including fatty acid metabolism, drug metabolism, oxidative phosphorylation, PPAR signaling, and TGF‐beta signaling. A risk prediction model based on the CoreGenes demonstrated high diagnostic value. Albumin (ALB), the liver's primary synthetic protein, has diagnostic value in chronic liver disease, though its specific role in NASH‐to‐HCC progression remains unclear. ALB dysfunction strongly correlates with liver damage. Chronic inflammation and oxidative stress lead to post‐translational modifications (PTMs) such as glycosylation and oxidation of ALB, thereby impairing its antioxidant and binding capabilities (Yang et al. 2021). Proteomics reveals inhibited fatty acid metabolism pathways in NASH liver tissue, contributing to impaired ALB function alongside hepatocyte damage (Lee et al. 2023). Although ALB itself is not directly carcinogenic, reduced binding capacity increases inflammatory factor concentrations (e.g., TGF‐β, IL‐6), activating the JAK/STAT pathway, stimulating HSCs, and promoting fibrosis (Febbraio et al. 2019; Vesting et al. 2022). BaP is metabolized by cytochrome P450 enzymes to form BPDE, which subsequently binds to plasma albumin to form BPDE‐albumin (BPDE‐Alb) adduct, causing DNA structural damage, increasing the risk of mutations, and ultimately leading to cancer (Guo et al. 2024).

Glutathione peroxidase 3 (GPX3) functions as a predominant extracellular antioxidant enzyme, maintaining intracellular redox homeostasis by scavenging reactive oxygen species (ROS), such as hydrogen peroxide (H_2_O_2_) and lipid peroxides. High‐fat diets in mice elevate 4‐HNE and reduce GPX3 in adipose tissue (Long et al. 2013), while BaP‐induced rat liver tumors show decreased GPX3 (Park et al. 2010). Hepatic GPX3 influences cell proliferation, apoptosis, and inflammatory responses by modulating the levels of ROS within the mitochondria and the cytoplasm. NASH progression involves ROS accumulation, promoting pro‐inflammatory factors (e.g., TNF‐α, IL‐6) and activating NF‐κB and JNK pathways, driving HSC activation and fibrosis.

Glutathione S‐Transferase Alpha 1 (GSTA1), a liver detoxification and antioxidant enzyme, regulates progression in various malignancies. Multiple cohort studies have confirmed that elevated GSTA1 expression is associated with prolonged overall survival in patients with NASH‐HCC (HR = 0.48, 95% CI: 0.32–0.72) (Liu et al. 2020). GSTA1 directly regulates lipid metabolism in NASH by interacting with fatty acid‐binding protein 1 (FABP1) and promoting its ubiquitination and degradation. This process inhibits intracellular triglyceride synthesis by impeding free fatty acid uptake and transport (Jiang et al. 2024).

Cytochrome P450 2E1 (CYP2E1), which is highly expressed in the liver, metabolizes endogenous and exogenous compounds, including drugs, toxins, and fatty acids. Its enzymatic activity promotes ROS accumulation, leading to cellular damage and chronic inflammation. CYP2E1 also plays a pivotal role in fatty acid β‐oxidation and peroxidation, producing lipid peroxides that promote liver fibrosis and accelerate NASH‐to‐HCC progression (Daly 2013; Jung et al. 2024; Sutti et al. 2014). Epoxide hydrolase 1 (EPHX1), an important hepatic biotransformation enzyme, is expressed at high levels in the liver. EPHX1 deficiency or dysfunction reduces antioxidant capacity, increases ROS accumulation, and activates pro‐carcinogenic pathways like NF‐κB and STAT3, promoting hepatocyte proliferation and survival. Dysregulated EPHX1 also disrupts carcinogen metabolism (e.g., 4‐HNE, BaP, PhIP), increasing NASH patients' HCC risk (Sun et al. 2021; Václavíková et al. 2015). Transferrin (TF) is a monomeric glycoprotein expressed and secreted by the liver into the plasma, which, together with transferrin receptors, is involved in iron transport. In tumor cells, transferrin participates in iron uptake, and both the transferrin receptor and GPX4 play critical roles in ferroptosis. Studies have shown that exposure to BaP markedly decreases TFRC levels, thereby inhibiting ferroptosis and promoting tumor cell invasion and migration (Wang et al. 2024). In addition, ectopic nuclear transferrin can enhance the hydroxylation of PPARα, thereby stimulating fatty acid oxidation and promoting hepatic tumor growth (Qian et al. 2025).

To assess the generalizability of our CoreGenes‐based diagnostic model, we performed external validation using the independent GSE162694 NASH cohort. The model demonstrated moderate to good discriminatory ability for advanced fibrosis (AUC = 0.802) and high NAS (AUC = 0.751). These results are slightly lower than the internal validation AUC of 0.967, which is expected given that external validation typically yields more conservative performance estimates due to cohort heterogeneity. Importantly, the significant association between risk scores and both fibrosis stage and NAS score indicates that our CoreGenes signature captures key pathological features driving NASH progression toward HCC, even in the absence of a dedicated NASH‐HCC validation cohort. These external validation results support the generalizability of our findings and the potential utility of the CoreGenes‐based risk model for risk stratification in NASH patients.

An intriguing finding emerged from our external validation that EPHX1 exhibited decreased expression in advanced fibrosis (F3–4) compared to mild fibrosis (F0–1) in the GSE162694 cohort, which contrasts with its upregulation in NASH‐HCC versus NASH in the GSE164760 dataset.

This apparent discrepancy may reflect the stage‐dependent dual role of EPHX1 in liver disease progression. During the fibrotic progression from mild to advanced NASH, downregulation of EPHX1 may indicate impaired detoxification and antioxidant capacity, as EPHX1 is known to play a critical role in cellular defense against xenobiotic toxicity and oxidative stress (Václavíková et al. 2015). Loss of EPHX1 function promotes oxidative stress and cellular damage, which could exacerbate liver injury and fibrosis. Conversely, during the transition from NASH to HCC, re‐upregulation of EPHX1 has been reported to activate pro‐carcinogenic signaling pathways, including JAK/STAT, promoting hepatocyte proliferation and survival (Xu et al. 2025). Such biphasic, context‐dependent roles are not uncommon in hepatocarcinogenesis—similar observations have been reported for other genes involved in xenobiotic metabolism and oxidative stress defense.

This finding underscores the complexity of EPHX1 biology and highlights the need for experimental validation across different stages of NASH‐HCC progression. Future studies using longitudinal cohorts or animal models spanning from NASH to HCC are warranted to dissect the temporal dynamics of EPHX1 expression and its functional implications.

Both innate and adaptive immune cells play critical roles in the initiation, progression, and malignant transformation of NASH to HCC. Our study illuminates the critical role of CoreGenes in reshaping the tumor immune microenvironment. In this study, we characterized the immune cell infiltration landscape in NASH and NASH‐HCC, revealing significant alterations in the abundance and composition of tumor‐infiltrating immune cells. Meanwhile, this study revealed that CoreGenes exerts a regulatory influence on the infiltration of specific immune cell types, including T cell regulatory, Macrophages M2, T cells CD8, Macrophages M1, and others. Regulatory T cells and M2 macrophages may promote carcinogenesis by suppressing immune responses and releasing pro‐tumor factors. CD8+ T cells can kill tumor cells by binding to them after recognizing tumor‐specific antigens (Pardoll 2012). In the tumor immune microenvironment, regulatory T cells may suppress the body's anti‐tumor immune response, leading to immune escape of tumor cells (Teixeira et al. 2022).

Our analysis revealed a dissociation between specific immune checkpoint expression and overall immune activity in NASH‐HCC tumors with high CoreGenes expression. Although CoreGenes expression levels were significantly negatively correlated with PD‐L1, PD‐1, and CTLA‐4, the IPS remained unchanged. This finding suggests that downregulation of these classical immune checkpoints does not necessarily lead to reduced overall immune activity in NASH‐HCC.

Immune cell infiltration analysis demonstrated that CoreGenes‐high NASH‐HCC tumors exhibited increased M1 macrophages, as well as decreased plasma cells, monocytes, M2 macrophages, and Tregs.

Collectively, CoreGenes‐high NASH‐HCC tumors display a distinct immune signature: despite reduced expression of classical immune checkpoint targets (PD‐L1/PD‐1/CTLA‐4) and altered immune cell composition, overall immune activity remains stable. This pattern suggests that resistance to anti‐PD‐1/PD‐L1 immunotherapy in NASH‐HCC patients with high CoreGenes expression may arise from target downregulation rather than systemic immunosuppression, accompanied by a remodeling of both innate and adaptive immunity. These results highlight the complexity of the tumor immune microenvironment in NASH‐HCC and suggest the existence of immune escape mechanisms beyond the classical PD‐1/PD‐L1 axis.

Genetic and epigenetic alterations in the coding gene, as well as dysregulation of microRNA (miRNA) activity, play a role in the development of HCC. To systematically elucidate how grilled food‐derived toxins (4‐HNE, BaP, and PhIP) exacerbate NASH‐HCC progression via epigenetic regulation, we constructed a comprehensive miRNA‐TF‐mRNA network. This network revealed that ALB and EPHX1, as key genes in glutathione metabolism, are directly regulated by the transcription factor FOXA1. Evidence has shown that the level of 4‐HNE in tumor tissues is associated with FOXA1 expression (Wang et al. 2021). Previous findings suggest that 4‐HNE may disrupt oxidative stress homeostasis through the transcription factor (TF)‐target gene axis, thereby exacerbating NASH progression. Furthermore, miRNAs including miR‐3130‐5p and miR‐153‐3p have been experimentally validated to regulate key transcription factors involved in HCC proliferation (Qi et al. 2020; Xu et al. 2024). miR‐30a‐3p levels were found to be upregulated in NAFLD patients' blood serum, which affected lipid deposition. In our regulatory network, these miRNAs directly target core genes (e.g., TF and GSTA1), indicating that grilled food toxicants (4‐HNE, BaP, and PhIP) likely promote NASH deterioration by interfering with epigenetic miRNA networks. This disruption impairs the expression of critical genes governing hepatic detoxification (GSTA1), oxidative stress defense (GPX3), and metabolic homeostasis (TF, CYP2E1), ultimately accelerating disease pathogenesis. This network reveals the “compounds‐TF/miRNA‐functional genes” regulatory relationship, validating computational predictions and providing a basis for miRNA‐based diagnostics and targeted interventions.

Molecular docking confirmed strong binding affinities of 4‐HNE, BaP, and PhIP to the core target proteins, providing a structural basis for their potential to directly disrupt protein function and contribute to toxicity. These findings elucidate the molecular mechanisms underlying the hepatotoxicity of grilled food‐derived compounds and provide a foundation for developing early warning biomarkers and targeted prevention strategies.

## 5 Conclusions

This study systematically deciphers the molecular mechanisms through which prominent grilled food toxicants (4‐HNE, BaP, PhIP) drive the progression from NASH to NASH‐HCC. We identified and validated a core signature of six genes (GSTA1, EPHX1, CYP2E1, GPX3, ALB, TF) as central players. These genes functionally converge on critical pathways including cellular oxidant detoxification, fatty acid metabolism (dysregulation), glutathione metabolism, PPAR signaling, and TGF‐beta signaling, creating a permissive environment for carcinogenesis. SHAP interpretability analysis highlighted ALB dysfunction (impaired binding capacity promoting lipotoxicity and inflammation) and reduced GPX3 (diminished antioxidant defense leading to ROS accumulation and signaling activation) as key contributors to progression risk. Molecular docking confirmed that these toxicants exhibit high‐affinity binding to core target proteins, suggesting a direct mechanism by which they may disrupt protein function and exacerbate toxicity. Moreover, exposure to these compounds promotes an immunosuppressive tumor microenvironment, marked by altered infiltration of Tregs and M2 macrophages. The robust diagnostic nomogram (AUC = 0.967) based on these CoreGenes offers significant clinical potential for early risk stratification. In summary, our findings provide a comprehensive mechanistic framework for grilled food toxicant‐induced NASH‐HCC progression and establish a translational foundation for targeted prevention strategies and early warning systems based on the CoreGenes signature. Future studies should prioritize in vivo validation of these targets and explore therapeutic interventions to mitigate their hepatotoxic effects, thereby informing more precise public health policies in food safety and environmental protection.

## 6 Limitations

Several limitations of this study should be acknowledged. First, while we performed external validation using an independent NASH fibrosis cohort (GSE162694), which confirmed the model's ability to stratify patients by fibrosis stage (AUC = 0.802) and NAS score (AUC = 0.751), the lack of a dedicated NASH‐HCC external cohort remains a limitation. The generalizability of our CoreGenes‐based diagnostic model to NASH‐HCC populations would benefit from further validation using additional cohorts with matched disease stages once such data become available. Second, all conclusions are derived from bioinformatics analyses and computational predictions, including network toxicology, machine learning, and molecular docking. No in vitro or in vivo experimental validation has been performed to confirm the functional roles of the identified CoreGenes or their interactions with the three toxicants. Finally, as a retrospective study based on publicly available data, the possibility of selection bias cannot be entirely excluded. Future studies should prioritize external validation using multi‐center NASH‐HCC cohorts and experimental validation to substantiate the mechanistic insights proposed in this study.

## Author Contributions

**Bingbing Qin:** conceptualization, methodology, data curation, formal analysis, project administration, visualization, writing – original draft, writing – review and editing. **Ye Zhang:** methodology, data curation, formal analysis, visualization, writing – review and editing. **Na Li:** methodology, data curation, writing – original draft, writing – review and editing, project administration. **Jing Li:** methodology, data curation, project administration, writing – original draft, writing – review and editing. **Yuehua Luo:** methodology, writing – review and editing. **Xi Tang:** methodology, writing – review and editing. **Ruisheng Zhou:** project administration, supervision, writing – review and editing. **Tianqi Cui:** methodology, data curation, writing – review and editing. **Ying Tang:** conceptualization, funding acquisition, project administration, supervision, writing – review and editing.

## Funding

This work was supported by the National Natural Science Foundation of China (Grant No. 82205220), the Science and Technology Projects in Guangzhou (Grant No. 2025A04J4355), and the State Key Laboratory of Traditional Chinese Medicine Syndrome Projects (Grant No. SKLKY2024B0013).

## Ethics Statement

The data used in this study, which came from publicly accessible databases containing records, did not necessitate ethical approval or informed consent.

## Conflicts of Interest

The authors declare no conflicts of interest.

## Supporting information


**Table S1:** Target genes of 4‐HNE, BaP, and PhIP.

**Table S2:** Differentially expressed genes in the GSE164760 dataset.

**Table S3:** Protein–protein interaction (PPI) network data of the DEGs.

**Table S4:** Genes selected by LASSO, SVM‐RFE, and Random Forest algorithms.

**Table S5:** miRNA‐TF‐mRNA interaction network of CoreGenes.

**Figure S1:** External validation of the CoreGenes‐based risk model in the GSE162694 cohort. (A) Gene expression by fibrosis groups: Bar chart showing core gene expression across fibrosis stages. (B) Gene‐fibrosis correlation: Scatter plots showing correlations between six core genes and fibrosis stages (F0‐F4). (C) Gene expression by fibrosis subgroups: Bar charts displaying each core gene's expression pattern across F0‐F4 stages. (D) Fibrosis severity validation: Left: Risk score comparison between mild (F0‐1) versus advanced (F3‐4) fibrosis. Right: ROC curve for fibrosis prediction. (E) NAS score validation: Left: Risk score comparison between low NAS (0–1) versus high NAS (6–7). Right: ROC curve for NAS prediction. *p < 0.05, **p < 0.01, ***p < 0.001.

**Figure S2:** Association of CoreGenes with immune checkpoint expression and IPS scores in NASH‐HCC. (A) Correlation analysis between CoreGenes expression and immune checkpoints. (B) Evaluation of the association between CoreGenes expression and IPS scores. *p < 0.05, **p < 0.01, ***p < 0.001.

## Data Availability

The datasets analyzed during the current study are publicly available in the Gene Expression Omnibus (GEO) repository under accession numbers GSE164760 and GSE162694.
